# Vestibular migraine: the most frequent entity of episodic vertigo

**DOI:** 10.1007/s00415-015-7905-2

**Published:** 2016-04-15

**Authors:** Marianne Dieterich, Mark Obermann, Nese Celebisoy

**Affiliations:** Department of Neurology, Ludwig-Maximilians University, Campus Grosshadern, Marchioninistr. 15, 81377 Munich, Germany; German Center for Vertigo and Balance Disorders, Ludwig-Maximilians University, Munich, Germany; Munich Cluster for Systems Neurology (SyNergy), Munich, Germany; Department of Neurology, University of Duisburg-Essen, Essen, Germany; Center for Neurology, Asklepios Hospitals Schildautal, Seesen, Germany; Department of Neurology, Ege University Medical School, Bornova, Izmir, Turkey

**Keywords:** Vestibular migraine, Episodic vertigo, Migrainous vertigo, Dizziness, International Headache Society, Bárány Society, Review

## Abstract

Vestibular migraine (VM) is the most common cause of episodic vertigo in adults as well as in children. The diagnostic criteria of the consensus document of the International Bárány Society for Neuro-Otology and the International Headache Society (2012) combine the typical signs and symptoms of migraine with the vestibular symptoms lasting 5 min to 72 h and exclusion criteria. Although VM accounts for 7 % of patients seen in dizziness clinics and 9 % of patients seen in headache clinics it is still underdiagnosed. This review provides an actual overview on the pathophysiology, the clinical characteristics to establish the diagnosis, the differential diagnosis, and the treatment of VM.

## Introduction

Symptoms of vertigo and headache are frequently observed by clinical neurologists. Since 1984 several studies have investigated the association of vestibular symptoms and migraine in adults [1–7]. Various terms have been used to describe this combination including migraine-associated vertigo, migraine-associated dizziness, migraine-related vestibulopathy, migrainous vertigo, and benign paroxysmal vertigo. To our knowledge, Dieterich and Brandt were the first to use the term ‘vestibular migraine’ (VM) [4]. VM is now the accepted name for vestibular symptoms that are causally related to migraine. The International Headache Society and the International Bárány Society for Neurootology have developed a consensus document with diagnostic criteria for VM [8]. This diagnosis was included in the appendix of the new international classification of headache disorders (ICHD)-3 beta version of headache classification as an emerging entity needing further research [9].

## Diagnostic criteria

The criteria for VM combine the typical signs and symptoms of migraine with the exclusion criteria of other disorders that also elicit vestibular signs (Table 1). As in migraine without aura, a diagnosis of VM mainly depends on the patient history, for so far there are no clinically useful biomarkers. The criteria of the consensus paper (Table 1) follow those established by Neuhauser and co-workers and validated during the last years for both ‘VM’ and ‘probable VM’ [5]. A positive predictive value of 85 % was found in a follow-up study conducted over 9 years [10]. The diagnosis described in the ICHD-3 beta version of the International Headache Society [9] closely approximates the criteria of migraine but requires that the vestibular symptoms last 5 min to 72 h for the diagnosis of VM.

**Table 1:** Vestibular migraine diagnostic criteria [8, 9]

A. At least five episodes fulfilling criteria C and D
B. A current or past history of migraine without aura or migraine with aura
C. Vestibular symptoms of moderate or severe intensity, lasting 5 min to 72 h
D. At least 50 % of episodes are associated with at least one of the following three migrainous features
Headache with at least two of the following four characteristics
Unilateral location
Pulsating quality
Moderate or severe intensity
Aggravation by routine physical activity
Photophobia and phonophobia
Visual aura
E. Not better accounted for by another ICHD-3 diagnosis or by another vestibular disorder

## Epidemiology and demographic factors

Case-controlled studies support the clinical association of migraine and vertigo revealing that migraine is more common in patients with vertigo than in age- and sex-matched controls [5, 11] and, also, that vertigo is more common in patients with migraine than in controls [1, 7, 12, 13].

Vestibular migraine is considered the most common cause of recurrent spontaneous vertigo attacks. It has a lifetime prevalence of about 1 % and a 1-year prevalence of 0.9 % in the general population [14] and accounts for about 7 % of patients seen in dizziness clinics and 9 % of patients seen in migraine clinics [5]. Nevertheless, it is still underdiagnosed. A recent study in a tertiary vertigo center found that the referring doctors had suspected only 1.8 % of the young patients to have VM, whereas a diagnosis was made in 20.2 % [15]. VM occurs 1.5 to 5 times more often in women than in men [3–5]. It has been proposed that VM has a genetic cause, namely an autosomal dominant pattern of inheritance with decreased penetrance in men [16].

While VM can develop at any age [2–4], it generally affects persons with a long-established history of migraine [4, 5]. It is diagnosed with an average delay of 8.4 years after the first onset of migraine [17]. The migraine attacks can be replaced by isolated vertigo attacks in postmenopausal women [18].

Epidemiological data confirm that migraine-related syndromes are also the most common cause of vertigo and dizziness in children [19, 20]. If the vertigo attacks in childhood take a monosymptomatic course without headache, they are called “benign paroxysmal vertigo in childhood”. The latter represents VM with aura but without headache. VM is with 39 % the most frequent form of vertigo in children followed by psychogenic/functional dizziness in 21 % [19]. The pediatric migraine variant of “benign paroxysmal vertigo in childhood” is characterized by brief attacks of vertigo associated with nystagmus that begin between the first and fourth year of life, last only seconds to minutes, and disappear spontaneously within a few years. It is benign and treatable. There are frequent transitions to other forms of migraine with and without aura.

## Clinical characteristics

### Symptoms

Spontaneous vertigo has been reported to occur in 21–83 % [2–4], positional vertigo and dizziness in 17–65 % [1, 4, 21], and head motion intolerance in 31–77 % of patients with VM [2, 3]. In a large population study based on telephone interviews, 67 % of the participants with VM reported spontaneous rotational vertigo, whereas 24 % had positional vertigo [14]. Vertigo has also been induced by moving visual objects [22]. In addition, in a study in a headache clinic the most common additional symptoms were unsteadiness (91 %), balance problems (82 %), and vertigo (57 %) [23]; these are vestibular symptoms that do not fulfill the diagnostic criteria of the International Bárány Society for VM [24].

Attack duration can vary from seconds to days [4, 5, 21]; however, the diagnostic criteria for VM require a 5-min minimum. Attacks lasting 5 to 60 min and fulfilling typical aura criteria were found in only 10–30 % of VM patients [4, 5], i.e., most patients did not meet the IHC criteria. An association of vestibular symptoms and headache is frequently seen, but it varies from patient to patient and from attack to attack, even in the same patient. Vertigo can precede or occur during or after headache [3, 5]. While less than 50 % have both symptoms in every attack, about 6 % report isolated vertigo attacks that alternate with migrainous headache symptoms [5]. Along with vertigo, patients may mention photophobia, phonophobia, osmophobia, visual and other auras that are relevant for a confirmation of the diagnosis. Auditory symptoms like hearing disturbances, tinnitus, and aural pressure have been found in 38 % of patients, but hearing is usually only mildly and transiently affected [1, 3, 21, 25].

### Clinical examination in the symptom-free interval

If a neurological examination is performed between the episodes, in the symptom-free interval, the findings are generally normal. However, central vestibular ocular motor abnormalities occur in 8.6 to 66 % of the patients [1–4, 26, 27] including gaze-induced nystagmus, saccadic pursuit, central positional nystagmus, dysmetric or slow saccades [4, 28]. A recent study showed that interictal ocular motor abnormalities increase over time, occurring in 16 to 41 % of patients during a follow-up of 5.5 to 11 years. The most frequent abnormality was central positional nystagmus [28].

Unilateral peripheral vestibular signs such as canal paresis have been reported in 8 to 22 % [1–4, 26, 27] and bilateral vestibular failure in up to 11 % [1, 3, 26]. Mild cochlear loss involving low frequencies has been documented in 3 to 12 % [1, 3, 29] and mild bilateral sensorineural hearing loss in 18 % in a follow-up study conducted over 9 years as a mean [28].

*During the acute attack* more patients (70 %) developed pathological nystagmus with either spontaneous or positional nystagmus [30]. Such findings made during the acute attack represent signs of a central vestibular dysfunction in 50 % and of a peripheral vestibular dysfunction in 15 %; the site of involvement was unclear in 35 %. Hearing was not affected in these patients [30].

### Neurophysiological testing

Vestibular migraine is a clinical diagnosis. Laboratory tests such as posturography, measurements of vestibular evoked myogenic potentials (VEMPs) and subjective visual vertical (SVV) have been used in different studies, but the results have been inconsistent. An increased postural sway was documented by posturography [26, 27]. Some studies reported that VEMPs were absent, delayed [31–33], or reduced in amplitude [31, 34, 35]. In contrast, other studies revealed symmetrical VEMPs with normal latencies and amplitudes [36, 37]. The measurements of SVV did not differ from those recorded in healthy controls [38].

## Pathophysiology

The mechanisms underlying vestibular dysfunction that are related to migraine still need further study and clarification. One explanation proposed is a parallel activation of vestibular and cranial nociceptive pathways [39–42]. Experimental studies have demonstrated that trigeminal and vestibular ganglion cells share neurochemical properties and express serotonin, capsaicin, and purinergic receptors [39, 43]. Nociceptive and vestibular afferents with neurochemical similarities converge in brainstem structures like the parabrachial nucleus, the raphe nuclei, and the locus coeruleus. All of these structures play an important role in modulating the sensitivity of pain pathways. They are also involved in the formation of anxiety responses, thus explaining some aspects of the comorbidity of balance disorders, anxiety, and migraine [41].

**Fig. 1:**
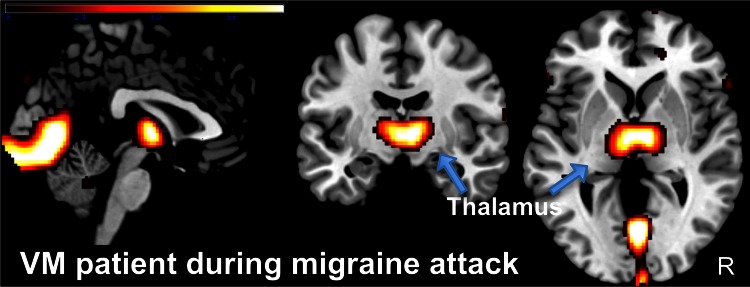
To analyze the cerebral blood glucose utilization during an actual VM attack a FDG-PET was performed in a 35-year-old patient suffering from VM according to the consensus criteria [8, 9] (ECAT Exact PET Scanner, Siemens/CTI, Knoxville, USA, with a 18F-fluorodeoxyglucose [FDG]-tracer in a three-dimensional acquisition mode). During the attack the patient presented with a central positional nystagmus beating oblique (up- and leftward) and increasing in different head/body positions (supine, left ear down, right ear down). Both, nystagmus and vertiginous sensation, persisted for 72 h and resolved spontaneously without any ongoing vestibular or ocular motor dysfunction. In addition, a structural T_1_-weighted MRI (MPRAGE sequence, 180 slices, slice thickness = 1 mm, image matrix = 256^2^, TR = 9.7 ms, TE = 4 ms) was acquired in a clinical 1.5 T scanner (Siemens Vision, Erlangen, Germany). The PET image was spatially normalised using the structural MRI data and a proportional scaling was performed to adjust for differences in tracer dosage and uptake time. A two-sample *t* test was computed with respect to a healthy, age-matched reference sample (*n* = 12) acquired on the same scanner under identical conditions (supine, eyes closed). During the attack the patient showed an increased cerebral glucose metabolism bilaterally in the ventral-anterior thalamus compared to healthy volunteers at rest (*p* < 0.001 uncorrected). The thalamic response was localized to the prefrontal thalamic projection zone [87]. The scale reflects the *z* score (personal communication: C. Best, Marburg, and P. zu Eulenburg, Mainz, Germany)

The cortical regions activated by vestibular stimulation in human functional imaging studies include those also involved in pain perception, for example, the posterior and anterior insula, the orbitofrontal cortex, and the cingulate gyrus [44–46]. A recent functional imaging study of two VM patients reported that the metabolism of the temporo-parietal-insular areas and bilateral thalami increased during the attack [45]. The cause was ascribed to increased activation of the vestibulo-thalamo-cortical pathways. Additional bilateral cerebellar activation was thought to be due to an adaptive process that suppresses the hyperactive vestibular system. A concurrent decrease in metabolism in the occipital cortex [47] was interpreted to represent the well-known reciprocal inhibition that occurs between the visual and vestibular systems [48]. A reciprocal inhibition of sensory cortex areas is typically involved in the intact sensory interaction occurring during vestibular stimulation [44, 48]. In an fMRI study of 12 right-handed VM patients during cold caloric stimulation a typical pattern of BOLD signal changes in temporo-parietal areas was found in the interictal interval as well as in patients with migraine without aura and in healthy controls [49]. In comparison to both control groups VM patients showed a significantly increased thalamic activation, the magnitude of which was positively correlated with the frequency of VM attacks. An increase of activity in the bilateral ventral-anterior thalamus was also seen in the FDG-PET during the VM attack compared to healthy controls at rest (personal communication, Fig. 1). Thus, the bilateral thalamus seems to play an important role in VM.

A voxel-based morphometric MRI study revealed that gray matter volume was reduced in areas associated with pain and visual and vestibular processing, i.e., in the superior, inferior and middle temporal gyri and in the mid cingulate, dorsolateral prefrontal, insula, parietal and occipital cortices. These areas possibly represent the pathoanatomic connection between the pain and the vestibular systems in migraine [50]. Thus, all these findings of the imaging studies indicate that there is a strong overlap of the vestibular and pain pathways at brainstem, thalamic, and cortical levels.

Reciprocal connections between the trigeminal and vestibular nuclei were identified in the one human study that has been performed [51]. It showed that trigeminal activation produced nystagmus in patients with migraine but not in healthy controls. This was attributed to a lowered threshold for signal transmission between the two systems. Various studies have discussed this feature, which indicates an increased vestibular excitability (hyperexcitability). Such an increase can include increased motion sensitivity, even motion sickness [52]; decreased suppression of the otoacoustic emissions [53]; and reduced perceptual thresholds of dynamic head movements [54]. The mechanisms underlying these changes still remain unclear.

Apart from central mechanisms an inner ear involvement may explain some cochlear and peripheral vestibular findings recorded in certain patients. Trigeminovascular reflex-mediated vasodilatation of cranial blood vessels and subsequently plasma extravasation causing meningeal inflammation are the key features of pain in migraine [55]. The trigeminovascular system also innervates the inner ear [56]. In line with this hypothesis, Koo and Balaban demonstrated a protein extravasation in the inner ear and meningeal tissues in a murine migraine model [57].

Similarities with other paroxysmal disorders that often present with both migraine and vertigo, for example, familial hemiplegic migraine and episodic ataxia type 2, have been reported to be associated with mutations in the calcium channel gene CACNA1A [58], and defects of the ion channels have also been discussed to play a role in VM [4]. So far, however, it has not been possible to identify a genetic defect in the same region [59, 60].

In summary, migraine-related vestibular disorders like VM may be caused by enhanced excitability occurring during the processing of sensory information, which is due to a genetic susceptibility. The enhanced excitation induces interactions of vestibular and pain pathways on several levels, from the inner ear to the thalamus and cortical level.

## Differential diagnosis/comorbidity

Ménière’s disease is the main differential diagnosis. At an early stage of the disease it may be difficult to differentiate Ménière’s disease from VM if aural symptoms are absent in Ménière’s disease. Even with the presence of aural symptoms it may be difficult since auditory symptoms like hearing disturbances, tinnitus, and aural pressure have also been found in 38 % of VM patients [1, 3, 21, 25]. To complicate matters, several studies have pointed to a link between Ménière’s disease and VM. The prevalence of migraine in patients with Ménière’s disease is reported to be twice as high as in healthy subjects, and the most reliable differentiating feature is the low-frequency hearing loss in Ménière’s disease [61]. A retrospective study showed that 13 % of patients fulfilled the criteria for both disorders, thus making the differential diagnosis even more complicated [25]. Indeed, an inner ear MR imaging study applying gadolinium-based contrast agent transtympanically showed an cochlear and vestibular endolymphatic hydrops in four of 19 VM patients (21 %) who presented with auditory symptoms [62]. This can either be explained by a coincidence of Ménière’s disease and VM or by the hypothesis that the hydrops is the consequence of a inner ear damage due to VM. Ménière’s disease and VM have also been considered part of a broad spectrum of disorders having a possible common genetic basis [63].

Benign paroxysmal positional vertigo (BPPV), for example, must also be considered in the differential diagnosis in those patients presenting with positional vertigo attacks, because BPPV is also commonly associated with migraine [64, 65].

Anxiety is a common comorbidity of migraine [66] and is frequently associated with vestibular disorders, especially with VM [67]. To define this association a new disorder named MARD (migraine–anxiety-related dizziness) has been proposed [68].

## Treatment

Only a few randomized controlled clinical studies have been conducted on the specific treatment of VM: during the attack or as prophylaxis. Two of these studies addressed the use of triptans for *attack therapy* [69, 70]. One study showed that 38 % of patients with VM attacks (3 of 8 episodes) benefitted from 5 mg zolmitriptan, whereas only 22 % in the placebo group (2 of 9 episodes) showed a positive effect. Unfortunately, the validity of this study is limited due to its large confidence intervals and the small number of patients (*n* = 10), who reported only 17 attacks [69]. The other double-blind, randomized, placebo-controlled study with rizatriptan vs. placebo measured how motion sickness responded to a complex vestibular stimulus. Twenty-five migraineurs with or without migraine-related dizziness participated (23 females; aged 21–45 years, 31.0 ± 7.8 years). Thirteen of the 15 subjects who experienced vestibular-induced motion sickness showed a decrease in motion sickness after taking rizatriptan compared to placebo (*p* < 0.02). However, this positive effect was not observed after exposure to more provocative vestibular stimuli. It was suggested that rizatriptan reduces vestibular-induced motion sickness by influencing serotonergic vestibular-autonomic projections [70].

*Prophylactic treatment* was analyzed recently in The Cochrane Collaboration [71] for randomized controlled trials in adults with the diagnosis of VM or probable VM according to the Bárány Society/International Headache Society criteria. Only 1 out of 558 studies could be identified which was based on the new criteria for VM and had adequate study conditions. This study comparing metoprolol and placebo is still ongoing [72]. Since none of the available studies to date are adequate, most therapeutic recommendations for the prophylactic treatment of VM are nowadays based on the therapy guidelines for migraine with and without aura. Therapeutic approaches that refer specifically to VM are found in case reports, retrospective cohort studies, and open-label trials.

A large retrospective cohort evaluation of 100 patients (median age 47 years, range 21–72 years) compared VM patients with and without prophylactic migraine treatment [73]. All patients on prophylactic treatment showed a decrease of duration, intensity, and frequency of episodic vertigo as well as its associated features (*p* < 0.01). The drugs taken were metoprolol (49 patients, 69 %; median dose 150 mg) or propranolol (31 %; median dose 160 mg), valproic acid (6 patients, 8 %; median dose 600 mg), topiramate (6 patients, 8 %; median dose 50 mg), butterbur extract (4 patients, 5 %; median dose 50 mg), lamotrigine (3 patients, 4 %; median dose 75 mg), amitriptyline (2 patients; 100 mg and 75 mg), flunarizine (1 patient; 5 mg), or magnesium (3 patients; median dose 400 mg). The group not receiving prophylactic therapy but instead following a modified lifestyle showed a reduction of only vertigo intensity [73]. Another retrospective study that included 100 patients with migraine-associated dizziness also reported a positive effect of migraine prophylaxis [74]. A third retrospective cohort included 33 patients with recurrent vertiginous attacks and migraine [75]: the attack frequency was completely reduced in 19 patients (57.6 %), reduced by over 50 % in 8 (24.2 %), and reduced by less than 50 % in 5 (15.2 %); there was no reduction in one patient. In this study 12 patients took propranolol, 11 received clonazepam, seven flunarizine, two metoprolol, and another two patients amitriptyline [75].

Smaller cohorts have reported on the effects of single drugs for migraine prophylaxis. *Sodium valproate* did not relieve the vestibular symptoms in a group of 12 patients with VM, but had a considerable effect on migraine headache in eight [76]. In this group the horizontal vestibulo-ocular reflex (VOR) was evaluated with the sinusoidal harmonic acceleration test at 0.01, 0.02, 0.04, 0.08, and 0.16 Hz using a computerized rotatory chair system. No abnormalities were found in VOR gain, phase, or asymmetry for any frequency. These normal VOR measurements contrasted with the repeated complaints by seven patients (58 %) of vertigo, dizziness, and unsteadiness, which valproate treatment did not improve [76].

*Cinnarizine* was tested in a retrospective, single-center, open-label investigation on VM and migraine associated with vertigo [77]. The study included 24 patients with VM (23 women, 1 man) and 16 patients with basilar-type migraine (12 women, 4 men). The patients’ ages ranged from 18 to 54 years (mean 30 years). The mean frequency of vertigo and also the mean frequency, duration, and intensity of migraine headaches per month were significantly reduced after 3 months of cinnarizine therapy (all *p* < 0.001) [77]. This interesting data will have to be reconfirmed in a large-scale, randomized, controlled clinical trial.

*Flunarizine* was tested for the treatment of migraine without aura and the treatment of vertigo in two large open-label post-marketing studies [78, 79]. In both conditions flunarizine showed considerable efficacy compared to propranolol for migraine headache or betahistine for vertigo. However, both studies did not specifically include patients with VM and thus the efficacy of flunarizine for this condition remains unproven. The only randomized controlled trial of one tertiary academic center compared the effects of flunarizine in 48 VM patients over 12 weeks with those receiving 16 mg betahistine and vestibular exercises [80]. The flunarizine treatment decreased the frequency of vertiginous episodes (*p* = 0.010), and the severity of vertigo improved (*p* = 0.046). However, frequency and severity of headache were not significantly different in the two treatment groups. Side effects of flunarizine were weight gain and somnolence [80]. A retrospective chart study evaluated the effects of flunarizine and propranolol in another 61 patients with VM. Flunarizine patients (*n* = 30) showed a 68 % responder rate for VM symptoms (*p* < 0.001), while patients on propranolol (*n* = 31) had an improvement rate of 73 % (*p* < 0.001) [81].

One trial reported successfully treating migraine auras, isolated auras, and to a lesser extent migraine-associated headaches with *lamotrigine* [82]. Another retrospective, open-label study demonstrated moderate efficacy of 100 mg lamotrigine in 19 VM patients (13 women, 6 men) over 3–4 months [83]. Vertigo frequency was reduced from 18.1 to 5.4 (average per month), headache frequency decreased from 8.7 to 4.4, but this was not statistically significant. Consequently, lamotrigine may primarily reduce vestibular symptoms but headache only to a less extent [83]. Lamotrigine was also reported useful in three patients with basilar-type migraine over 5 years [84].

An interesting study investigated the combination of effects resulting from the abstinence from *caffeine* and treatment with topiramate and nortriptyline in 34 VM patients [85]. The symptoms were improved in 14 % of the patients who had abstained from caffeine. In comparison, topiramate reduced symptoms in 25 % of patients and nortriptyline reduced dizziness in 46 % of the patients (*p* = 0.007). Thus, 75 % of VM patients had a measurable and meaningful benefit from these therapeutic interventions; consequently they did not switch to other treatments [85].

Less established medications in migraine treatment such as benzodiazepines, selective serotonin reuptake inhibitors (SSRI), pizotifen, dothiepin, acetazolamide, and behavioral modification including special diets were reported to have positive effects on VM [75]. However, a clear therapeutic recommendation for the specific treatment of VM cannot be easily drawn from these data. Moreover, it must be taken into account that inconsistent definitions of VM were used in many of these studies especially in the older ones, so that the examined cohorts were quite heterogeneous. The new diagnostic criteria will eliminate this obvious shortcoming in the future and lead to more comparable, better quality studies.

*Vestibular rehabilitation training* proved effective in VM patients as add-on treatment to medical therapy or as a stand-alone treatment option [86]. Thirty-six patients (VM = 20, vestibular impairment  = 16) with daily vestibular symptoms participated in a 9-week vestibular rehabilitation program. Each patient attended five therapy sessions over 6 months. While the VM group demonstrated poorer subjective performance at therapy onset, both groups benefitted equally from rehabilitation. The same degree of improvement was observed in the migraine group regardless of the medication regime. Thus, vestibular rehabilitation training may be effective in VM regardless of the medical prophylactic therapy used [86]. This agrees with the well-known positive effect of physical activity on the reduction of migraine attack frequency. However, a study with a controlled design is still needed for VM.

The *future perspectives* of both clinical and basic science studies investigating the pathophysiological mechanisms of VM are promising. Understanding the neurochemical organization of the vestibular, nociceptive, and cognitive pathways and their interactions will provide realistic strategies for treatment of the disorder. Further research is needed to clarify the probable genetic mechanisms leading to greater susceptibility. Multicenter randomized controlled treatment trials based on pathophysiology must now be designed on the basis of the recently established diagnostic criteria.
